# AutoDTI++: deep unsupervised learning for DTI prediction by autoencoders

**DOI:** 10.1186/s12859-021-04127-2

**Published:** 2021-04-20

**Authors:** Seyedeh Zahra Sajadi, Mohammad Ali Zare Chahooki, Sajjad Gharaghani, Karim Abbasi

**Affiliations:** 1 Department of Computer Engineering, Yazd University, Yazd, Iran; 2 Laboratory of Bioinformatics and Drug Design (LBD), Institute of Biochemistry and Biophysics, University of Tehran, Tehran, Iran

**Keywords:** Drug-target interactions, Deep learning, Unsupervised learning, Latent feature, Denoising autoencoder

## Abstract

**Background:**

Drug–target interaction (DTI) plays a vital role in drug discovery. Identifying drug–target interactions related to wet-lab experiments are costly, laborious, and time-consuming. Therefore, computational methods to predict drug–target interactions are an essential task in the drug discovery process. Meanwhile, computational methods can reduce search space by proposing potential drugs already validated on wet-lab experiments. Recently, deep learning-based methods in drug-target interaction prediction have gotten more attention. Traditionally, DTI prediction methods' performance heavily depends on additional information, such as protein sequence and molecular structure of the drug, as well as deep supervised learning.

**Results:**

This paper proposes a method based on deep unsupervised learning for drug-target interaction prediction called AutoDTI++. The proposed method includes three steps. The first step is to pre-process the interaction matrix. Since the interaction matrix is sparse, we solved the sparsity of the interaction matrix with drug fingerprints. Then, in the second step, the AutoDTI approach is introduced. In the third step, we post-preprocess the output of the AutoDTI model.

**Conclusions:**

Experimental results have shown that we were able to improve the prediction performance. To this end, the proposed method has been compared to other algorithms using the same reference datasets. The proposed method indicates that the experimental results of running five repetitions of tenfold cross-validation on golden standard datasets (Nuclear Receptors, GPCRs, Ion channels, and Enzymes) achieve good performance with high accuracy.

## Background

Protein targets are strictly related to some diseases. The target’s biological activities reveal due to the therapeutic impact of drugs on these diseases. Therefore, to animate or repress a target’s biological process in the drug discovery process, we consider a drug's interaction with the target proteins [1]. Thus, drug–target interactions (DTIs) play a prominent role in drug discovery. However, identifying and validating drug candidates via biological assays, from introducing the abstract concept to release it into the market, usually take 10–15 years and costs 0.8–1.5 billion dollars [2]. Therefore, various computational methods to predict drug–target interactions are being used to aid the drug discovery process. Computational methods have some advantages, including low drug development costs, short time, low drug safety risk, and exploring a wide range of potential drug–target interactions. The computational approaches received more attention in recent years. Chen et al. [3], for DTI prediction, introduced some state-of-the-art computational models, including network-based approach and machine learning-based approach. Bagherian et al. [4] described data and databases required and broad category consisting of a machine learning approach for DTI prediction. Ding et al. [5] concentrated on machine learning-based methods, especially similarity-based methods that use drug and target similarities. Abbasi et al. [6] reviewed the deep learning-based approach in DTI, and they give some perspective on the future approaches.

In DTI prediction, computational approaches are divided into three major groups. The first group is called the ligand-based approach, which uses similar molecules and the similarity between the target proteins’ ligands [7]. However, the results obtained from ligand-based methods might be incorrect when the number of target’s known ligands are insufficient [8]. The second group comprises the docking approach. In this approach, the 3D structures of drug and protein are taken into account and used to determine their interaction tendency. One of the limitations of this approach is that they require the 3D structure of the target proteins [9, 10]. Hence, these methods could not be applied to new drug-target pairs that the 3D structures of proteins are unavailable [11]. For example, predicting the 3D structure for targets like GPCRs is still challenging [12]. The third group comprises the chemogenomics approaches that utilize information of drug and target concurrently to predict DTI. One of the advantages of chemogenomics approaches is that many online public databases can access their available data. For example, information such as the genomic sequences of targets and the chemical structure of drugs are used for DTI prediction [13]. This approach doesn't have the limitations mentioned in the previous two groups. The chemogenomics approach usually uses machine learning and deep learning methods for DTI predictions. This paper concentrates on computational methods that belong to the chemogenomics approach.

The proposed method by Chen et al. [14] integrated three different networks, such as protein–protein similarity network, drug-drug similarity network, and known drug-target interaction networks, into a heterogeneous network by known drug–target interactions and performed the random walk on this heterogeneous network. Mazharul Islam et al. [15] proposed a DTI-SNNFRA framework for DTI prediction based on shared nearest neighbor (SNN) by a partitioning clustering for sampling the search space in the first stage and fuzzy-rough approximation (FRA) in the second stage. Zeng et al. [16] proposed a network-based deep-learning method for DTI prediction by integrating ten networks called DeepDR. Then the low-dimensional representation of drugs and drug-disease pairs by a variational autoencoder were learned from the heterogeneous networks. Lim et al. [17] introduced a novel approach for predicting DTI based on a graph neural network that directly organized the 3D structural information on a protein–ligand binding posed into an adjacency matrix. A distance-aware graph attention mechanism was also devised to increase the performance of the model. Zong et al. [18] proposed a DeepWalk deep learning method for drug-target interaction prediction based on network topology similarity measures. Firstly, a heterogeneous network created from biomedical linked datasets. After that DeepWalk was selected to measure the similarities within linked tripartite network (LTN).

With the increase of experimental data, the use of deep learning methods to predict DTIs has been increasing. Deep learning methods learn the input data's hierarchical features, leading to better performance than other standard machine learning methods. In deep learning-based DTI prediction, a drug-target pair has taken as input, and then the affinity of interaction is predicted as output. Wen et al. [19] adapted a deep learning method named DeepDTI that used a deep belief network (DBN). Their approach predicted the affinity value for a pair of FDA-approved drugs and targets. In their work, protein targets were not divided into different classes. The features of drugs were automatically extracted from extended-connectivity fingerprints (ECFP), and the features of target proteins were extracted from the composition of amino acids, dipeptides, and tripeptides [20]. Peng et al. [21] used sparse autoencoders to reduce the original features' dimension into a hidden representation, and then they trained a support vector machine (SVM) with hidden representation. In another study called DL-CPI [22], which used protein domain information, domain binary vectors were employed to represent the domains used to describe proteins. Ozturk et al. [23] introduced a DTI prediction approach which used the convolutional neural network (CNN) to learn the feature vectors for drug and protein target. On a kinase family bioassay dataset, their approach performed better [24, 25] than the conventional models like kronRLS-MKL [26] and SimBoost [27]. In a paper by Lee et al. [1], their DeepConv-DTI model predicted massive-scale DTIs using raw protein sequences for various target protein classes and diverse protein lengths. New protein features were generated with convolution filters on the entire protein sequence to capture local residue patterns. Then protein features and the drug features were concatenated and fed into the subsequent layers to predict the affinity value. Finally, their model was optimized with DTIs from MATADOR [28]. Abbasi et al. [29] combined convolutional layers and Long Short-Term Memory (LSTM) layers to learn more effective local substructures through a compound and a protein. Then they utilized a two-sided attention mechanism to weight each local substructure of the compound and protein sequence.

As an unsupervised approach to DTI prediction, matrix factorization (MF) techniques learn the latent feature matrices of drugs and targets from the DTI matrix. These two latent feature matrices are multiplied to reconstruct the interaction matrix for prediction. Among various unsupervised methods in DTI, regularized matrix factorization methods achieve a higher performance among the previous DTI prediction methods [30, 31]. Matrix factorization techniques suffer from the cold start problem as well as the sparsity. In this study, to overcome the issues mentioned above, the unsupervised approach of deep learning is utilized to extract latent factors of input data. To this end, in this paper, we have developed a new drug-target interaction prediction method named AutoDTI++, an unsupervised deep learning model by using denoising autoencoder. Denoising autoencoder is an unsupervised deep neural network that learns the latent factors from the matrix interaction. However, the learned latent factors are not very effective due to the sparse nature of the drug-target interaction matrix. Additional information such as drug fingerprints information has been utilized to address the drug-target interaction matrix sparsity problem.

To evaluate our proposed method, we have used cross-validation to compare it with six other state-of-the-art methods, namely DDR [32], DNILMF [33], NRLMF [34], KronRLS-MKL [26], BLM-NII [35], and COSINE [36]. We have evaluated the ability of AutoDTI++ using new drug cross-validation, new interaction cross-validation, and new target cross-validation. We computationally simulated a new target case and a new drug case (by leaving out their respective interactions) and tested our proposed method on these cases to investigate its ability to predict the left-out interactions. Finally, our model achieved better performance than most previous models.

In section methods, firstly, we describe the dataset used in our work in “Dataset” section. Our notations are described in “Notations” section. An overview is done on the neural network of denoising autoencoder (DAE) in “Denoising autoencoder” section. Then, our proposed method is described in “Workflow” section. The experimental results of our work, relevant discussion, and conclusion are given in the next sections, respectively.

## Methods

### Dataset

This study used the introduced benchmark dataset in [9] to evaluate our proposed approach. This dataset contains four different target protein types, namely nuclear receptors (NR), G protein-coupled receptors (GPCR), ion channels (IC), and enzymes (E). Table 1 shows some statistics, including the number of unique proteins, number of unique drugs, number of interactions, and the sparsity ratio for each dataset. The variable $Y\in {\mathbb{R}}^{n\times m}$ denotes the interaction matrix where *n* represents the number of drugs and *m* denotes the number of targets. Suppose the drug ${d}_{i}$ and the target ${t}_{j}$ interact, then ${Y}_{ij}=1$, otherwise ${Y}_{ij}=0$. Rows and columns of Y show the profiles of drugs and targets, respectively. The interaction profile for each drug or target is determined by ${Y}_{d}$ and ${Y}_{t}$, respectively. Sparsity denotes the ratio between the number of DTIs and the number of all possible DTIs.

**Table 1:** Drugs, targets, interactions, and sparsity in each dataset

Datasets	NR	GPCR	IC	E
No. of drugs	54	223	210	445
No. of targets	26	95	204	664
No. of interactions	90	635	1476	2926
Sparsity	0.064	0.030	0.034	0.01

## Preliminaries

In this section, first, we define the notations used in this paper. Then, we simply introduce denoising autoencoder.

## Notations

The notation used in this paper is listed as follows:

${Y}_{d}$, ${Y}_{t}$ are the sparse row/columns of $Y$

${\tilde{Y }}_{d}$, ${\tilde{Y }}_{t}$ are corrupted versions of ${Y}_{d}$, ${Y}_{t}$

${\widehat{Y}}_{d}$, ${\widehat{Y}}_{t}$ are dense estimates of ${Y}_{d}$, ${Y}_{t}$

${\overline{Y }}_{d}$, ${\overline{Y }}_{t}$ are dense low-rank representations of ${Y}_{d}$, ${Y}_{t}$

### Denoising autoencoder

An autoencoder is an unsupervised neural network that includes two networks: an encoder and a decoder aiming to reconstruct the input domain. The encoding network maps the input to a hidden representation [37]. The decoding network reconstructs the original inputs from the hidden representation [38]. As a result, autoencoder is used to learn feature representation in an unsupervised manner. An autoencoder is considered a neural network that obtains higher-level representations of input data without requiring ground-truth label information. Given a training sample $x$ ($x\in {R}^{{d}_{0}}$), it is encoded into the hidden representation $y\in {R}^{{d}_{1}}$ by the mapping ${f}_{c}$:

1 $$\mathbf{E}\mathbf{n}\mathbf{c}\mathbf{o}\mathbf{d}\mathbf{e}\mathbf{r}: y={f}_{c}\left(x\right)={S}_{c}({V}^{T}x+{b}_{1})$$

where ${S}_{c}$ is the non-linear activation function of the encoder. Also, $V$ and ${b}_{1}$ are respectively the weight matrix and the bias vector. After that, the representation of the hidden layer *y* is mapped to the reconstructed output ${x}^{^{\prime}}$ of the same shape as $x$ by function ${f}_{d}$:

2 $${\mathbf{D}\mathbf{e}\mathbf{c}\mathbf{o}\mathbf{d}\mathbf{e}\mathbf{r}: x}^{^{\prime}}={f}_{d}\left(y\right)={S}_{d}\left({W}^{T}y+{b}_{2}\right)$$

where ${S}_{d}$, W, and ${b}_{2}$ are the same parameters of the decoder network. The full autoencoder is indicated by ${\varvec{n}}{\varvec{n}}\left({\varvec{x}}\right)\stackrel{\scriptscriptstyle\mathrm{def}}{=}{{\varvec{f}}}_{{\varvec{d}}}\left({{\varvec{f}}}_{{\varvec{c}}}\left({\varvec{x}}\right)\right)$**.**

Recently, many autoencoders have been introduced, like denoising autoencoder, sparse autoencoder, and variational autoencoder [29]. Denoising autoencoders add some noise to the input and then force the network to reconstruct the denoised input. One method to add some noise is to mask a random fraction of the input by replacing them with zero. In this case, we use the modified loss function to emphasize the denoising aspect of the network. To this end, two weight hyperparameters $\alpha$ and $\beta$ are used to weight the terms as follows:

3 $${L}_{\alpha ,\beta }\left(x,\tilde{x }\right)=\alpha \left(\sum_{j\epsilon \mathcal{C}\left(\tilde{x }\right)}{\left[nn{\left(\tilde{x }\right)}_{j}-{x}_{j}\right]}^{2}\right)+\beta \left(\sum_{j\notin \mathcal{C}\left(\tilde{x }\right)}{\left[{nn\left(\tilde{x }\right)}_{j}-{x}_{j}\right]}^{2}\right)$$

where $\tilde{x } \in {\mathbb{R}}^{N}$ is a corrupted version of the input $x$, $\mathcal{C}$ is the set of corrupted elements in $\tilde{x }$, $0<\alpha ,\beta <1$, and ${nn\left(x\right)}_{j}$ is the ${j }^{th}$ the output of the network while fed with $x$.

## Workflow

In this section, the proposed drug-target interaction prediction method called AutoDTI++ is presented, which consists of three steps:

(i) The first step includes a pre-processing step that transforms the binary values in the given drug-target matrix, Y, into the binary values in the drug fingerprint-target interaction matrix for filling missing values based on drug fingerprint.
(ii) The second step is to propose an AutoDTI model that uses an unsupervised deep learning technique based on denoising autoencoders to predict drug–target interactions.
(iii) The third step includes a post-processing step in which the drug-target interaction matrix is predicted from the output of the second step.

After presenting these three steps, we will present the proposed approach.

### Pre-processing step

While deep learning has many successes in image and speech recognition [39], sparse data has received less attention and remains a challenging problem for neural networks. Therefore, there is no standard approach for using the sparse matrix as inputs of deep neural networks yet. Most papers on sparse inputs are obtained by pre-calculating estimates of missing values [40]. Sparse inputs have already been studied in the industry [41], where 5% of the values are missing. However, datasets in DTI often face more than 95% missing values. Since the drug-target interaction matrix relies on only interactions between drugs and targets, when additional information is available for the drugs and the targets, only using the interaction matrix can sound restrictive. Therefore in our case, we want to handle this issue by adding information on drugs fingerprint to the interaction matrix. Our approach uses the fingerprint of drugs to handle autoencoders' sparse input. To this end, the following steps are done:

1. The first step represents the drug molecule by SMILES (simplified molecular-input line-entry system): each drug is represented by SMILES [42] strings, a sequential encoding of chemical structures.
2. The second step, create the fingerprint-drug matrix (Z): utilize the PaDEL-descriptor software to transform SMILES string to fingerprints. PaDEL-descriptor software is used for calculating molecular descriptors (1D, 2D descriptors, and 3D descriptors) and ten types of fingerprints [43]. Each drug can be represented as a binary vector with a length of 800, in which indices indicate the existence of the specific substructures.
3. The third step, create the fingerprint-target matrix (W = $Z.Y$): We multiply the fingerprint-drug matrix (Z) by the drug-target interaction matrix (Y). The result is a fingerprint-target matrix (W).
4. The fourth step, normalization: normalize the fingerprint-target matrix with the min–max method.
5. The fifth step, convert to the binary matrix: Since values greater than zero in this matrix represent an interaction between the target and the drug fingerprint, these values are replaced by one.

By performing these five steps, the obtained matrix is not sparse like the raw drug-target interaction matrix. With these pre-processing steps, almost half of the fingerprint–target interactions matrix is known.

### The AutoDTI model

In the AutoDTI model, if it is assumed that the model's input is a drug-target interaction matrix, then drug-target known interactions can be encoded as a partially drug-target interaction matrix Y $$\in {\mathbb{R}}^{n\times m}$$. Each drug $$d\in D=\left\{1\dots n\right\}$$ can be represented by a partially observed vector $${Y}_{d}=\left({Y}_{d1},\dots {Y}_{dm}\right)\in {\mathbb{R}}^{m}$$. Similarly, each target $$t\in T=\left\{1\dots m\right\}$$ can be represented by a partially observed vector $${Y}_{t}=\left({Y}_{1t},\dots {Y}_{nt}\right)\in {\mathbb{R}}^{n}$$. Our aim in this work is to design a drug-based (target-based) autoencoder which can take each partially observed $${Y}_{d}$$ ($${Y}_{t})$$ as input, project it into a low-dimensional latent space and then reconstruct $${Y}_{d}$$ ($${Y}_{t}$$) in the output space to predict unknown interactions. We reconstruct the sparse vectors $${Y}_{d} \left({Y}_{t}\right)$$, into dense vectors $${\widehat{Y}}_{d}\left( \widehat{{Y}_{t}}\right)$$. In this case, it is needed to define two types of autoencoders:

- D-AutoDTI is defined as ${\widehat{Y}}_{d} \stackrel{\scriptscriptstyle\mathrm{def}}{=}nn\left({Y}_{d}\right)$
- T-AutoDTI is defined as ${\widehat{Y}}_{t}\stackrel{\scriptscriptstyle\mathrm{def}}{=}nn\left({Y}_{t}\right)$

The learned parameters are regularized to prevent the over-fitting of the observed interactions. Formally, the objective function for the D-AutoDTI model is:

4 $$\underset{\theta }{\mathrm{min}}\sum_{d=1}^{n}{\Vert {Y}_{d}-nn\left({Y}_{d},\theta \right)\Vert }_{o}^{2} +\frac{\lambda }{2}\cdot \left({\Vert W\Vert }_{F}^{2}+{\Vert V\Vert }_{F}^{2}\right)$$

where ${\Vert .\Vert }_{F}^{2}$ means that we only consider the contribution of the known interactions and regularization strength $\lambda$ > 0. The proposed approach's training loss differs from the classic autoencoders, which only aim to reconstruct the input. Given the learned parameters$\widehat{\theta }$, D-AutoDTI's predicted interactions for drug d and target t are:

5 $${\widehat{Y}}_{dt}={\left(nn\left({Y}_{d};\widehat{\theta }\right)\right)}_{t}$$

**Fig. 1:**
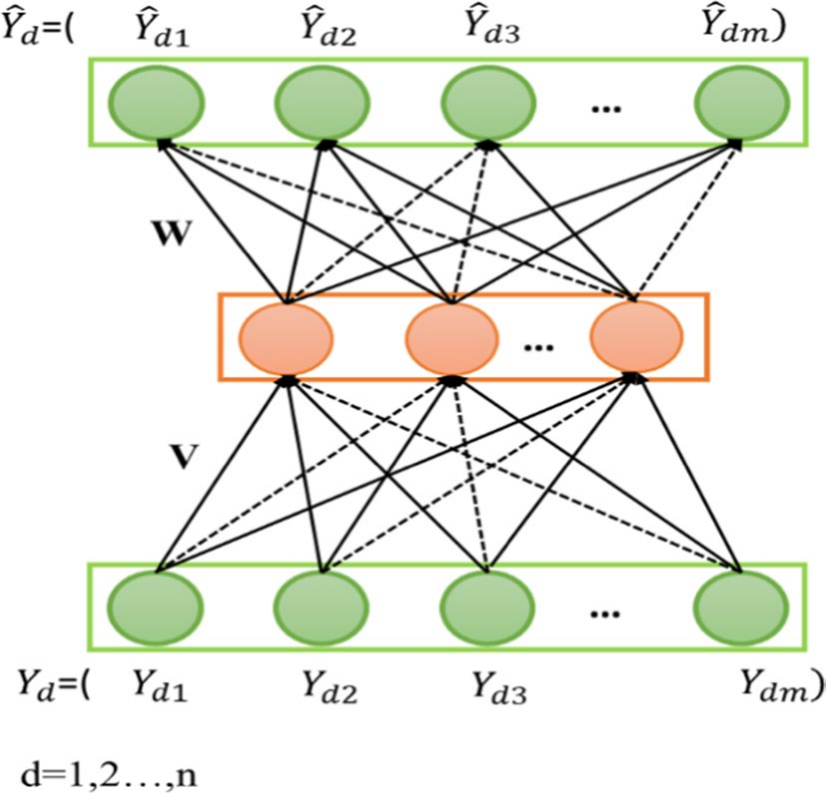
The overall schematic of the D-AutoDTI model. The shaded nodes show the known interactions, and the solid connections show the weights that are updated for the input ${{\varvec{Y}}}_{{\varvec{d}}}$. There are n copies of the neural network for each drug

Figure 1 shows the overall schematic of the utilized autoencoder. The shaded nodes illustrate the known interactions, and the solid connections show the weights that are updated for the input ${Y}_{d}$.

To train the autoencoders, the following three steps are performed:

i) Assign zero to unknown interactions in the edges of input layers,
ii) back-propagated values in the edges of the output layers are replaced by zero values,
iii) use a denoising loss to emphasize interaction prediction over interaction reconstruction.

One way to restrain the edges of the input is to turn the missing values to zero. We utilize an empirical loss that ignores the loss of unknown values to preserve the autoencoder from always returning zero. Missing values do not bring information to the network. The error is discarded for missing values. Therefore, the empirical loss back-propagates the error for known values while no error is back-propagated for missing values. In other words, this operation is equivalent to removing the neurons with missing values described in [44, 45]. Finally, masking noise is used from the denoising autoencoders empirical loss. Autoencoders in the training process are trained to predict missing values by simulating them. The final target is the prediction of these missing values. Thus, the classic unsupervised training of autoencoders converts to simulated supervised learning by emphasizing the prediction criterion. The training can be turned into pseudo-semi-supervised learning by mixing both criteria of reconstruction and prediction. The denoising autoencoders’ loss becomes an assuring objective function. The final training loss function after regularization is:

6 $${L}_{\alpha ,\beta }\left({Y}_{d},{\tilde{Y }}_{d}\right)=\alpha \left(\sum_{j\epsilon \mathcal{C}\left({\tilde{Y }}_{d}\right) }{\Vert {\left({Y}_{d}\right)}_{j}-{nn\left({\tilde{Y }}_{d}\right)}_{j}\Vert }_{o}^{2}\right)+\beta \left(\sum_{j\notin \mathcal{C}\left({\tilde{Y }}_{d}\right) }{\Vert {\left({Y}_{d}\right)}_{j}-{nn\left({\tilde{Y }}_{d}\right)}_{j}\Vert }_{o}^{2}\right)+\frac{\lambda }{2}\cdot \left({\Vert W\Vert }_{2}^{f}+{\Vert V\Vert }_{2}^{f}\right)$$

**Fig. 2:**
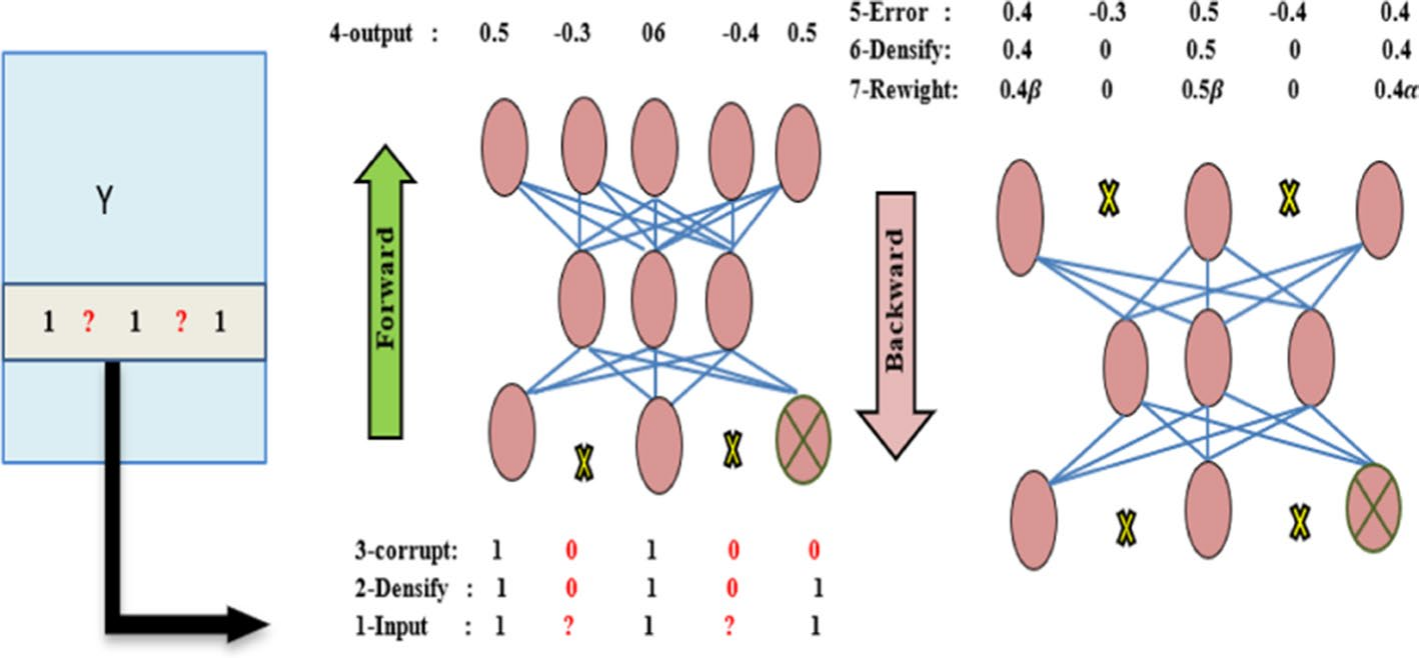
Feed-Forward/Backward process is shown for denoising autoencoder. The input is obtained from the matrix of interactions, unknown values are turned to zero, some interactions input are corrupted, and a dense estimate is finally constructed. Before back-propagation, unknown interactions are converted to zero error. Use ${\varvec{\beta}},\boldsymbol{ }\boldsymbol{\alpha }$ hyper-parameters, reconstruction, and prediction errors are reweighed

W and V are the vectors of weights of the network, and $\lambda$ is the regularization hyper-parameter. The full-forward/backward process is explained in Fig. 2.

### Post-processing

In the post-processing step, the drug-fingerprint matrix (${Z}^{T}$) is multiplied by the output of the AutoDTI model ($\widehat{W}$). The product of multiplication is equivalent to the predicted drug-target interaction matrix.

### AutoDTI++ proposed method

**Fig. 3:**
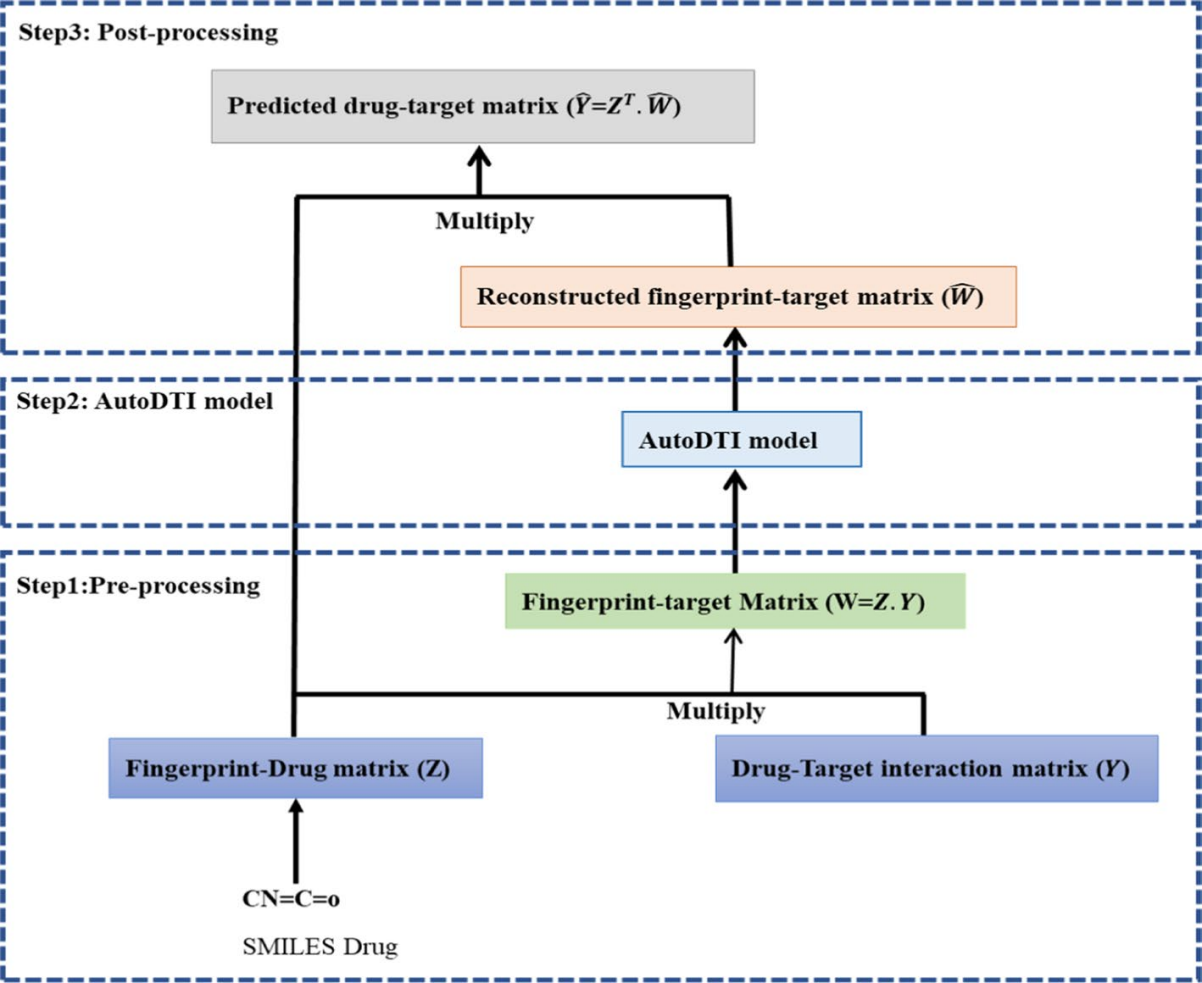
The AutoDTI+ +  method includes three steps: pre-processing, AutoDTI model, post-processing

As shown in Fig. 3, the AutoDTI++ proposed method is performed in three steps which include: the first step is pre-processing, which explained in “Pre-processing step” section. The second step uses the AutoDTI model explained in “The AutoDTI model” section. In AutoDTI ++ proposed method, the fingerprint-target matrix is applied as the AutoDTI model input instead of the drug-target interaction matrix. The third step is post-processing that explained in “Post-processing” section. Fingerprint-target reconstructed matrix ($\widehat{W}$) is calculated as follows:

7 $$\mathrm{W}=Z\cdot Y$$

8 $$\widehat{W}={\varvec{n}}{\varvec{n}}\left(W;\widehat{\theta }\right)$$

Z is a fingerprint-drug matrix, and Y is a drug-target matrix. Predicted interactions of AutoDTI ++ for drug d and target t are:

9 $${\widehat{Y}}_{dt}={{Z}^{T}}_{d}\cdot{\widehat{W}}_{t}$$

where ${{Z}^{T}}_{d}$ is ${d}^{th}$ row of ${Z}^{T}$ matrix, ${\widehat{W}}_{t}$ is ${t}^{th}$ the column of W reconstructed matrix.

## Results

First, we introduce the cross-validation (CV) and the metric we used to evaluate our models. Second, we present the parameter settings. Then, we present some baseline approaches which are compared with our model. Finally, we compare our models with the baselines to illustrate the performance of our model.

### Cross-validation experiments

We performed cross-validation under three scenarios described in [46] to perform a comprehensive empirical comparison among various methods as follows:

(1)${S}_{p}$, denote the random drug–target pairs that are left out to be used as the test set;

(2)${S}_{d}$, denote the entire drug interaction profiles that are left out to be used as the test set; and.

(3)${S}_{t}$, denote the entire target interaction profiles that are left out to be used as the test set.

${S}_{p}$ is the traditional method for performance evaluation. Meanwhile, various approaches to predict interactions for new drugs and targets are evaluated using ${S}_{d}$, and ${S}_{t}$ test sets. Here, new drugs and targets are those for which no interaction information is available in the training set. As such, conducting experiments under ${S}_{d}$ and ${S}_{t}$ provides information about the proposed approach's generalizability.

Such as previous works, we employed the area under the receiver operating characteristic (AUC) curve and the area under the precision-recall (AUPR) curve to evaluate prediction performance. We performed experiments to compare our proposed method with the existing techniques, including DDR, DNILMF, NRLMF, KRONRLS-MKL, BLM-NII, and COSINE. Specifically, we conducted five repetitions of the tenfold CV for each of the methods under each of the above scenarios using AUPR [47] as the evaluation metric. That is, the interaction data set was divided into ten folds, and each fold, in turn, was left out as the test set while the remaining nine folds were treated as the training set. The prediction performance for each of the folds is evaluated in terms of AUPR. This process is repeated five times, and the final AUPR score was the average over five such repetitions. For all experiments, AUPR was used as the main metric for performance evaluation. AUPR is more adequate because it heavily penalizes incorrect predictions of interactions [48], which is desirable here. After all, we do not want false predictions recommended by the prediction algorithm in practice.

### Parameter settings

Experiments are conducted on the benchmark database [9]. We repeated this splitting procedure 5 times and reported average AUPR and AUC. First, we calculated AUC and AUPR on NR, GPCR, IC, and E datasets for the AutoDTI method without pre-processing. The obtained results are not acceptable. Then, we applied a pre-processing step on the AutoDTI method and called that AutoDTI++ . Interestingly, after a pre-processing step, AutoDTI significantly improved the results of AUC and AUPR on all datasets.

We evaluated the performance of the AutoDTI++ model as the number of hidden units and the number of hidden layers varied. We observed that performance steadily increases with two hidden layers of (15, 5) units. We used sigmoid activation functions in each layer. Using a non-linear activation function in the hidden layer is critical for the excellent performance of AutoDTI ++. We did fine-tuning by gradient-based back-propagation with a minibatch of size 100. We set the regularization strength to 10 for IC, GPCR, and E datasets, and we set it to 1 for the NR dataset.

**Impact of the loss:** we investigated the effects of hyper-parameters $\alpha ,\beta$ on denoising loss. To this end, we used a greedy search, and the best performance is achieved with $\alpha =0.4$ and $\beta$=0.6.

### Comparisons with the state-of-the-art algorithms

**Fig. 4:**
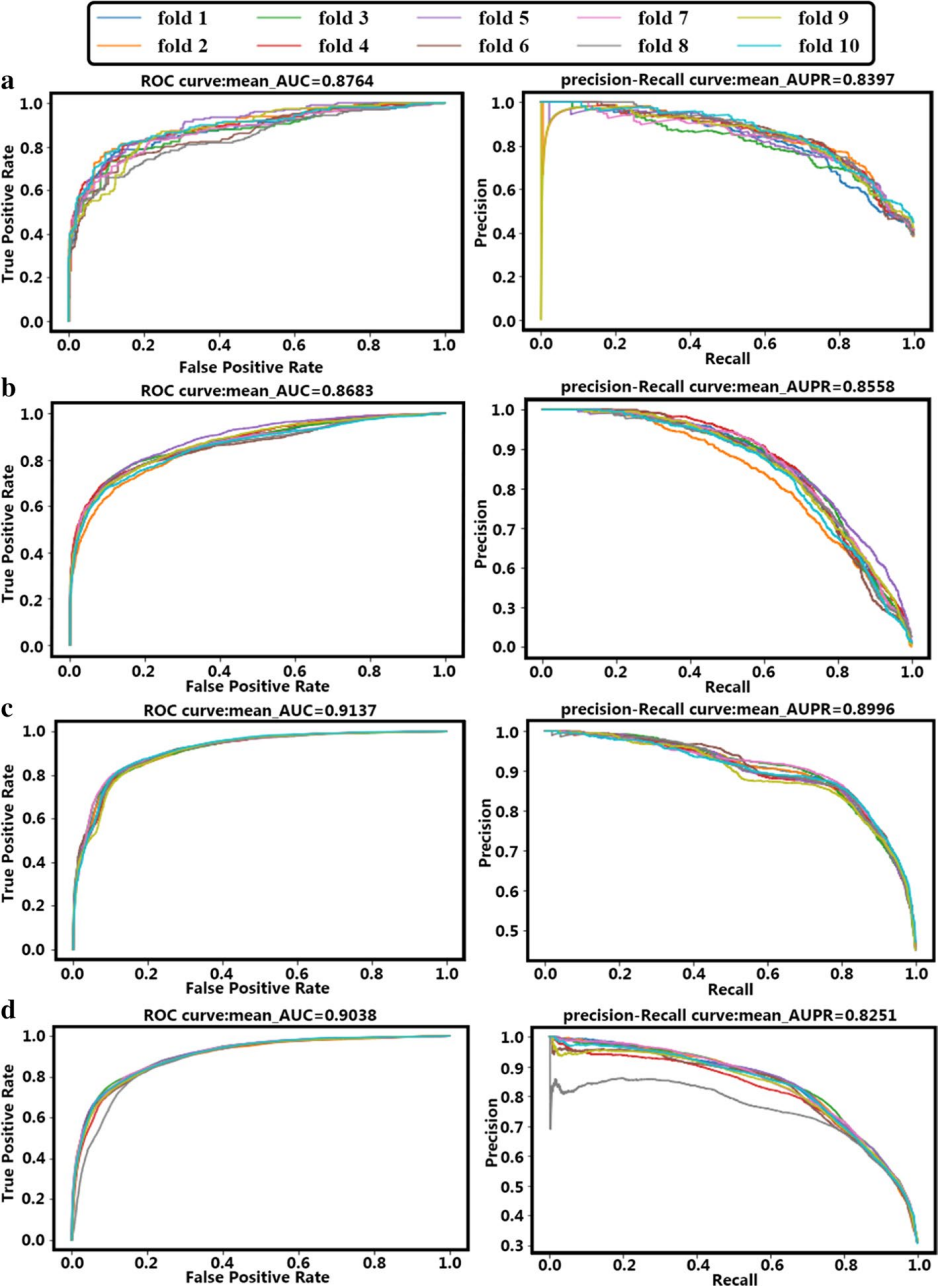
The ROC and precision-recall curves of the first repeat of tenfold CV for four datasets; the right side is the precision-recall curve, and the left is the ROC curve. **a** The precision-recall and ROC curves for NR dataset; **b** The precision-recall and ROC curves for GPCR dataset; **c** The precision-recall and ROC curves for IC dataset; **d** The precision-recall and ROC curves for E dataset

AutoDTI++ method calculates AUC and AUPR on NR, GPCR, IC, and E datasets. For NR, GPCR, IC, and E datasets, AUPR and AUC scores for ${S}_{p}$,${S}_{d}$, and ${S}_{t}$ test sets show in Table 2. Figure 4 shows the ROC curve and precision-recall curve of the first repeat of tenfold cross-validation on four datasets. The mean-AUC and mean-AUPR are the average AUC and average AUPR of AutoDTI ++ in the first repeat of tenfold CV.

**Table 2:** AUC and AUPR scores of AutoDTI ++ approach obtained under three prediction tasks (${S}_{p}$, ${S}_{d}$, and ${S}_{t}$) overall datasets (NR, GPCR, IC, and E) by 5 repeats of tenfold CV

AutoDTI ++	NR	GPCR	IC	E
$$\bf \bf {S}_{p}$$				
AUPR	0.84	0.85	0.90	0.82
AUC	0.87	0.86	0.91	0.90
$$\bf \bf {S}_{d}$$				
AUPR	0.62	0.47	0.50	0.33
AUC	0.60	0.47	0.49	0.50
$$\bf \bf {S}_{t}$$				
AUPR	0.84	0.83	0.86	0.77
AUC	0.87	0.85	0.86	0.84

#### Baseline approaches

To measure the prediction performance, six existing state-of-the-art DTI prediction methods are used to compare with our AutoDTI++ model on NR, GPCR, IC, and E datasets under three different CV settings, including DDR, DNILMF, NRLMF, KronRLS-MKL, and BLM-NII, and COSINE.

#### DDR

First, it is based on using a heterogeneous graph that applies a similarity selection procedure to select a set of informative and less-redundant similarities for drugs and target proteins. DDR combines different similarities using the non-linear similarity fusion method. Then, manually, 12 different path-category-based feature patterns from the heterogeneous network are extracted. Finally, DDR applies a random forest model to predict DTIs.

#### KronRLS-MKL

First, it applies the weighted combination of multiple drug kernels and target kernels to get the final drug kernel and target kernel, and then KronRLS uses Kronecker product algebraic properties as the drug-target pairwise kernel. Finally, it uses Kronecker regularized least squares to predict DTIs.

#### NRLMF

NRLMF method focuses on modeling the probability. The interaction probability of a drug with a target is calculated by a logistic function of the drug-specific and target-specific latent vectors. Furthermore, the neighborhood regularization based on the local structure of the drug-target interaction data is utilized to improve the model's prediction ability.

#### DNILMF

DNILMF method is followed by the non-linear combination technique of multiple similarity measures for drugs and target proteins, as well as smoothing new drug-target predictions based on their neighbors.

#### BLM-NII

in BLM–NII, the neighbor-based interaction-profile inferring (NII) procedure is integrated into the bipartite local model (BLM) framework to form a DTI prediction approach, where the RLS classifier with GIP kernel was used as the local model.

**Fig. 5:**
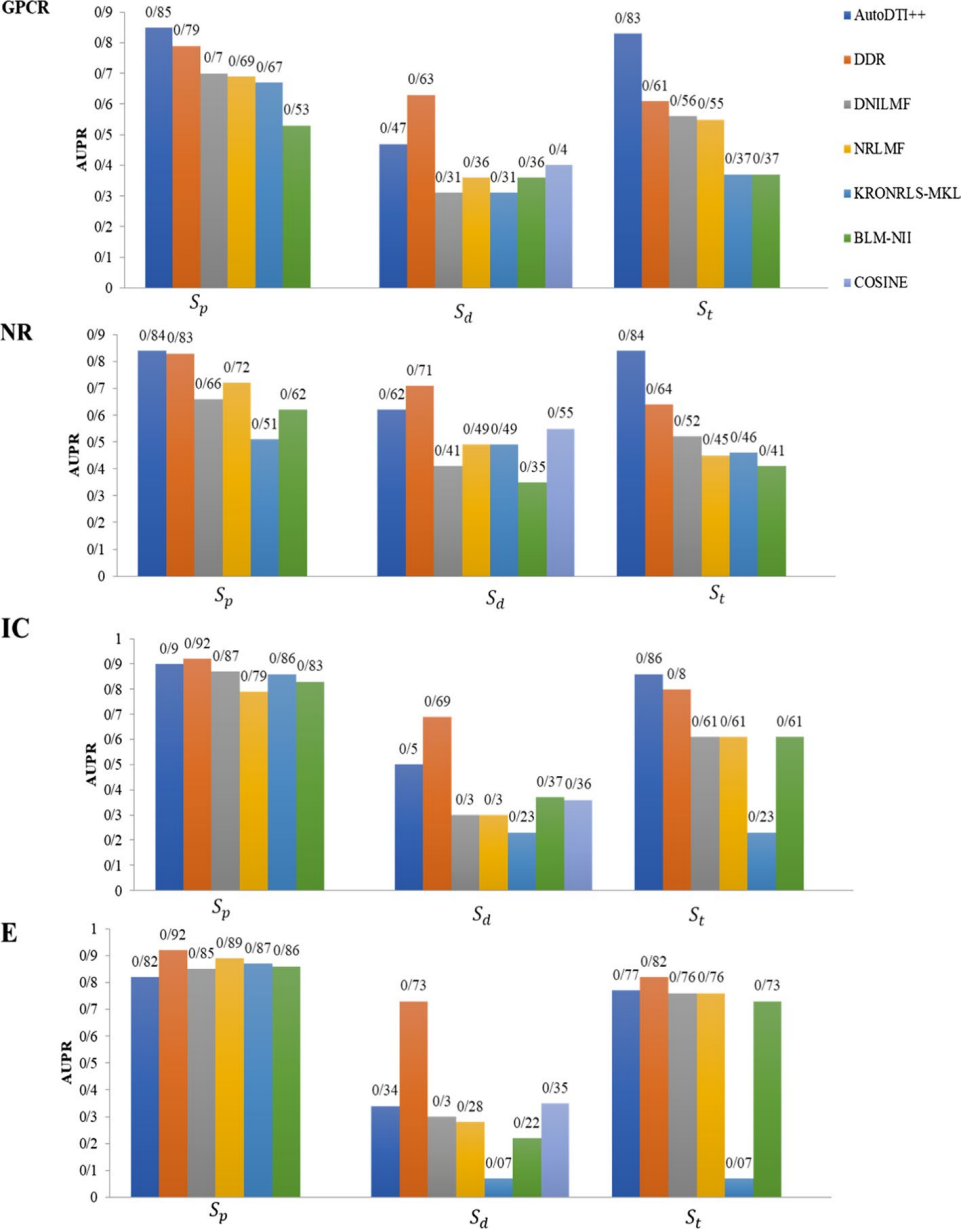
Comparison results of AutoDTI++ method with the six states-of-the-art methods (DDR, DNILMF, NRLMF, KRONRLS-MKL, BLM-NII, and COSINE) in terms of AUPR scores, using 5-repeats of tenfold CV. Results are obtained under ${S}_{p}$, ${S}_{d}$, and ${S}_{t}$ settings on NR, GPCR, IC, and E datasets. The results for DDR, DNILMF, NRLMF, KRONRLS-MKL, BLM-NII, and COSINE are captured using the best parameters published

We used 5-repeats of tenfold cross-validation to evaluate the predictive performance of DDR, KronRLS-MKL, NRLMF, DNILMF, BLM-NII, and COSINE for comparison with the AutoDTI++ method under the ${S}_{p}$ CV setting. Figure 5 shows the comparison AUPR of AutoDTI++, DDR, KronRLS-MKL, NRLMF, DNILMF, BLM-NII, and COSINE on four datasets under the ${S}_{p}$ CV setting.

We have shown that AutoDTI++, using 5-repeats of tenfold CV, achieves acceptable AUPR values than the other methods. From Fig. 5, we can see that, in terms of AUPR, under ${S}_{t}$ setting on three datasets of NR, GPCR, and IC, the performance of the AutoDTI++ model is improved. The AutoDTI++ model on NR, GPCRs, and IC data sets performs better than DDR that is the best baseline method. The AutoDTI++ model, on the E dataset, performs better than all approaches except the DDR method. AutoDTI++ model achieves results for NR, GPCR, and IC, which respectively are 20%, 22%, and 6% higher than DDR. In terms of AUPR, under ${S}_{d}$ the setting, the AutoDTI++ model is better than all other approaches except the DDR approach on all datasets. In terms of AUPR, under ${S}_{p}$ the setting, the AutoDTI++ model performs better than DDR on NR and GPCRs datasets. AutoDTI++ model achieves results for NR and GPCR which are 1% and 6%, higher than DDR but for E and IC datasets, DDR method which are 10% and 2%, higher than AutoDTI++.

## Case study

To evaluate the practical ability of AutoDTI++, we applied it to predict novel DTIs that are unknown in NR, GPCR, IC, and E datasets. For the prediction of novel interactions, we applied the trained model in all datasets. Then we used from the output the interaction probability. The predicted probability is ranked in descending order. The high-probability drug-target pairs are predicted as novel DTIs in NR, GPCR, IC, and E datasets. We selected the top-ranked unknown DTI interaction for each dataset. To validate these new interactions, we selected several reference databases that included ChEMBL [49], DrugBank [50], KEGG [51], CTD [52], and STITCH [53]. These reference databases included many validated known DTIs obtained from experimental and published results on drug–target interactions.

The CTD reference database found drug D00217 represents acetaminophen, strongly inhibiting the enzyme cytochrome P450 2C8. AutoDTI++ also identified an interaction between D00217 and hsa1558 without a known interaction in the E dataset.

The KEGG reference database found drug D00636 that represents amiodarone hydrochloride, strongly inhibiting the target sodium voltage-gated channel alpha subunit 5. AutoDTI++ also identified the interaction between D00636 and hsa6331 without a known interaction in the IC dataset.

The DrugBank reference database found drug D02340 that represents loxapine, strongly inhibited the target dopamine receptor D1. AutoDTI++ also identified the interaction between D02340 and hsa1812 without a known interaction in the GPCR dataset.

In the ChEMBL reference database, found drug D00585 represents mifepristone strongly inhibited the target estrogen receptor 1. AutoDTI++ also identified the interaction between D00585 and hsa2099 without a known interaction in the NR dataset.

## Discussion

This study introduces a novel DTI prediction method, AutoDTI++, which utilizes a denoising autoencoder for DTI prediction using a drug fingerprint-target interaction matrix. We have shown that we can achieve a more accurate prediction for different datasets by pre-processing the drug-target interaction matrix and applying it to the AutoDTI prediction model. To evaluate the proposed work, on different representative datasets, under various cross-validation settings, and using AUPR and AUC as the performance measures, we have shown that AutoDTI ++ outperforms the other state-of-the-art methods that we used in the comparison. We also demonstrated that AutoDTI++ performs significantly better than the other existing methods when known DTIs are missing in the training data. We can see that AutoDTI performs worse because of the lack of additional side information and sparsity of the interaction matrix. In the proposed method, we used the drug fingerprint, which analyzes molecules as a graph and retrieves the molecular substructures from the whole molecular graph's subgraphs. Specifically, we used PaDEL-descriptor to extract a fingerprint from a raw SMILES string. Finally, each drug can be represented as a binary vector with a length of 800 whose indices indicate specific substructures' existence. In our model, the drug fingerprint provides additional information to build an interaction matrix without sparsity. Actually, if a drug interacts with a target, that target probably interacts with the substructure of that drug. Therefore, if the drug-target matrix, which is a sparse matrix, is multiplied by the drug-fingerprint matrix, which contains the drug substructure and is non-sparse, is obtained the fingerprint-target matrix, which is a non-sparse matrix and solves the problem of the sparse interaction matrix. Also, drug fingerprint adds additional information to the interaction matrix to build a more accurate model. Therefore, the AutoDTI++ model can handle the sparse interaction matrix and learn a much more effective feature vector for each drug, and our proposed model achieves much better performance. We observed that the best second method in predicting DTI in the ${S}_{p}$ and ${S}_{t}$ cross-validation settings and the first method in ${S}_{d}$ cross-validation setting, in terms of the AUPR metric over the four different datasets, is the DDR method. The DDR approach utilizes a heterogeneous drug–target graph that contains information about various similarities between drugs and similarities between proteins as drug targets. The DDR gives better results than the AutoDTI++ model, in the ${S}_{d}$ setting. Possibly, one reason is that it uses the similarity between drugs while smoothing the predictions of new drugs by incorporating neighbor information based on the assumption that similarity may contribute to the accuracy of the predictions for their neighbors. As a result, the DDR model achieves better results in ${S}_{d}$ cross-validation setting.

Approaches based on MF (NRLMF, DNILMF) perform worse than the AutoDTI++ model, especially in AUPR. Possibly, one reason is that AutoDTI++ can learn a non-linear latent representation through sigmoid activation function while MF models learn a linear latent representation. Therefore our proposed method learns sufficient and effective features by autoencoders neural networks to detect true DTIs. Also, a good advantage of using autoencoders in the AutoDTI++ approach is that they can fill in every vector that is not present in training data that leads to the superiority of the AuoDTI++ over the MF method. Another reason might be that MF approaches embed both drugs and targets into a shared latent space, but the AutoDTI++ model only embeds the target into latent space and uses the drug fingerprint feature.

In terms of AUPR, AutoDTI++ performs on IC better than E, NR, and GPCR datasets, possibly because IC has less sparsity than other datasets on matrix interaction. GPCR and NR have sparsity approximately the same, but NR is a little better than GPCRs, possibly because the number of targets affects results. Regarding a dataset, the input vector with a less number of targets is more suitable. Because the input vector with a larger number of targets is more sparsity difference, that results in an imbalance model. E dataset performs wost than other datasets because it has more sparsity in between all datasets.

## Conclusions

We proposed a novel method called AutoDTI++ to predict DTIs based on autoencoders. Our proposed approach includes three steps. The first step consists of a pre-processing step that transforms the binary values in the given drug-target matrix to the binary values in the drug fingerprint-target interaction matrix for filling missing values based on drug fingerprint. The second step proposed an AutoDTI model that uses an unsupervised deep learning technique based on denoising autoencoders to predict interactions, and the third step is post-preprocessing. Subsequent pre-processing is applied to the AutoDTI model, and it achieves better performance. Experimental results show that the AutoDTI++ model achieves significantly more accurate results than the other state-of-the-art methods under cross-validations ${S}_{p}$, ${S}_{d}$, and ${S}_{t}$ on NR, GPCR, IC, and E datasets, and different metrics of performance evaluation. As future work, first, we plan to expand our model by adding some additional information, such as amino acid sequences of target proteins. Second, we will develop our models to incorporate some additional information, such as similarity drugs and targets matrix, to solve the interaction matrix's sparsity problem. Finally, we will combine our models with other models of autoencoders.

## Data Availability

The datasets used in this project can be found in http://web.kuicr.kyoto-u.ac.jp/supp/yoshi/drugtarget/.

## Abbreviations

DTI: Drug-target interaction
CV: Cross-validation
MF: Matrix factorization
DBN: Deep belief network
SVM: Support vector machine
ECFP: Extended-connectivity fingerprints
CNN: Convolutional neural network
DAE: Denoising autoencoder
NR: Namely nuclear receptors
GPCR: G protein-coupled receptors
IC: Ion channels
E: Enzymes
AUC: Area under the receiver operating characteristic curve
ROC: Receiver operating characteristic
AUPR: Area under precision-recall curve
NII: Neighbor-based interaction-profile inferring
BLM: Bipartite local model
LTN: Linked tripartite network
SNN: Shared nearest neighbor
FRA: Fuzzy-rough approximation
LSTM: Long short-term memory
